# *Cassia fistula*: A remedy from Traditional Persian Medicine for treatment of cutaneous lesions of pemphigus vulgaris

**Published:** 2017

**Authors:** Fatemeh Atarzadeh, Mohammad Kamalinejad, Ladan Dastgheib, Gholamreza Amin, Amir Mohammad Jaladat, Majid Nimrouzi

**Affiliations:** 1 *Department of Traditional Iranian Medicine, School of Traditional Medicine, Tehran University of Medical Sciences, Tehran, Iran *; 2 *School of Pharmacy, Shaheed Beheshti University of Medical Sciences, Tehran, Iran*; 3 *Molecular Dermatology Research Center, Department of Dermatology, Shiraz University of Medical Sciences, Shiraz, Iran*; 4 *Department of Traditional Pharmacy, Tehran University of Medical Sciences, Tehran, Iran *; 5 *Department of Traditional Persian Medicine, School of Medicine, Shiraz University of Medical Sciences, Shiraz, Iran*

## Abstract

**Objective:**

Pemphigus is a rare autoimmune disease that may be fatal without proper medical intervention. It is a blistering disease that involves both the skin and mucus membranes, in which the most important causes of death comprise superimposed opportunistic infections and complications of long-term high-dose corticosteroid therapy or prolonged consumption of immune suppressant drugs. Skin lesions are the most important sources of infection, and any local treatment decreasing the healing time of lesions and reducing the total dosage of drugs is favorable.

**Materials and Methods:**

Here, we review the probable mechanism of action of a traditional formulary of *Cassia fistula* (*C. fistula*) fruit extract in almond oil as a new topical medication for reducing the duration of treatment of pemphigus vulgaris erosions.

**Results:**

*C. fistula *fruit oil has lupeol, anthraquinone compounds as rhein and flavonoids.

Previous *in vitro* and animal studies on *C. fistula* fruit have demonstrated wound healing, antioxidative, anti-leukotrienes, anti-inflammatory, antibacterial and antifungal effects of this plant.

**Conclusion:**

It is hypothesized that *C. fistula* L. can be a botanical therapeutic choice for treatment of pemphigus erosions.

## Introduction

Pemphigus is a rare autoimmune disease that may be fatal without proper medical intervention. It is a blistering disease that involves both the skin and mucus membranes (Bystryn and Rudolph, 2005 ▶). While pemphigus may affect everyone all over the world (Kershenovich et al., 2014 ▶; Ruocco et al., 2013b ▶), females are more prone to the disease (Chams‐Davatchi et al., 2005 ▶). The most common form of pemphigus is pemphigus vulgaris (PV). Skin blisters usually appear weeks and months after the appearance of painful oral erosions. Flaccid blisters develop gradually on erythematous or even normal skin, and they may be localized in one area for a long time. Blisters, if left untreated, always become generalized. The blisters tear easily and painful pruritic erosions are the common finding. However, the healing process progresses very slowly (Ruocco et al., 2013a ▶) and the delayed healing process is substantial either due to its psychological effects or treatment costs imposed by hospitalization. A study on the quality of life (QoL) of patients with pemphigus, showed a strong impact of the disease on patients physical and emotional status as they were more worried or depressed than the general population (Paradisi et al., 2009 ▶).

There is no international consensus among experts regarding its treatment modality (Habif, 2009). The aim of pemphigus management is to induce and maintain remission. This entails suppression of blister formation, healing of erosions, and ultimately withdrawal of treatment (Martin et al., 2011 ▶). From 1950 to 1969, treatment of patients with PV essentially comprised of systemic administration of corticosteroids. However, the side effects of these drugs became a major cause of morbidity and mortality (Poulin et al., 1984 ▶). The most important causes of death are superimposed opportunistic infections and complications of long-term high-dose corticosteroid therapy or prolonged consumption of immune suppressant drugs (Ahmed, 2001 ▶). 

The 5-year mortality rate of pemphigus was about 100% before the advent of effective medications such as systemic corticosteroids. Currently, the use of immunosuppressive and systemic corticosteroid drugs has decreased the mortality rate of the disease to about 5-15% which is mostly related to the side effects of these medications (Iraji and Banan, 2010 ▶). 

Review studies on PV during 2011 and 2012 show that conclusive clinical evidence regarding interventions for PV is inadequate. Optimal therapeutic strategy for PV has remained unclear and further research is needed (Kasperkiewicz et al., 2012 ▶).

Traditional and complementary medicines are sources of new and usually natural remedies. Among various traditional systems of medicine, Traditional Persian Medicine (TPM) is one of the oldest and most valuable ones (Zargaran et al., 2013 ▶) and has the potency of introducing medicinal plants that can be used as an adjuvant therapy for pemphigus erosions.

In this regard, we aimed to introduce a traditional Iranian candidate, named *Cassia fistula* L*. *(*C. fistula*) for pemphigus erosion. This herbal drug has been mentioned in many traditional Iranian medicinal books as a therapeutic choice for mouth ulcers and skin inflammations similar to PV lesions. Possible mechanisms of action were investigated by searching PubMed, Google scholar and Elsevier databases.

To the best of our knowledge, there is no clinical trial or even case report about the wound healing effects of herbal drugs in pemphigus; thus, our review suggests a better strategy for wound healing in pemphigus.

## Materials and Methods

We first searched for recent ethiopathogenesis findings about PV emerging in PubMed and Google Scholar. Then, PV lesions were considered as “hot ulcers” in TPM according to clinical presentation. Afterwards, the botanical management of “hot ulcers” was searched in major TPM sources, two important clinical textbooks of TPM, namely Canon of Medicine by Avicenna (10th and 11th centuries), and Eksir-e-Aazam (The Great Elexir) by Mohammad Azam Khan (19th century) as well as two comprehensive TPM pharmacopeias, namely Tohfatol Moemenin (The Present for the Faithfuls) by Mohammad Tonkaboni (17th century), and Makhzanol Advieh (The Storehouse of Medicaments) by Aghili-Shirazi (18th century). Finally, the available data from recent articles on the therapeutic mechanisms of action of *C. fistula* were compared with those from TPM textbooks.

**Pathophysiology of pemphigus**

Desmogleins (Dsg) are cell-to-cell adhesion molecules contained in desmosomes. Circulating IgG autoantibodies are directed against the normal Dsg proteins within the desmosomal structure on the cell surface of keratinocytes. The autoantibodies destroy the adhesion between epidermal cells (Habif, 2009). Involvement of T helper (Th) cells in the pathogenesis of PV has been suggested by several studies. A critical role was suggested for auto-reactive T cells in the induction and regulation of antibody production (Veldman et al., 2003). Several cytokines have been linked to PV pathogenesis, including interleukin-4 (IL-4), IL-5, IL-6, and IL-10, which suggests Th2 involvement. Th1 cells also seem to be necessary for developing PV. Depletion of CD4+ T cells has been shown to decrease serum levels of anti-Dsg3 antibodies in PV patients. Strong evidence has shown that B cells are activated by Dsg3-specific auto-reactive T cells through IL-4 secretion (Okon and Werth, 2014 ▶).

 Oxidative stress seems to be responsible for the onset/aggravation of many human diseases including pemphigus (Abida et al., 2012 ▶). Reactive oxygen species (ROS) have an important role in the inflammation process. Increased production of ROS in PV leads to a decline in antioxidants level in plasma and red blood cells which results in oxidative stress. Inflammatory pathways lead to neutrophil activation and release of ROS, which mostly implicate their adverse effect through inflammatory cytokines, lipid peroxidation and abscission of dermal-epidermal junction in PV (Yesilova et al., 2013 ▶).

There are significant correlations between serum oxidative stress marker levels and serum anti-desmoglein antibody levels in pemphigus. This enforces the implication of oxidative stress in the pathophysiology of pemphigus via an increase in the autoantibodies’ reactivity (Abida et al., 2012 ▶).

 Leukotrienes are abundantly expressed in the skin in diverse inflammatory skin diseases including PV and have been suggested to contribute to the pathogenesis of pemphigus. Moreover, these results indicate that different classes of leukotrienes play distinct and essential roles in this disease. Leukotriene B4 and C4 levels in human skin are highly elevated under diverse inflammatory skin conditions including psoriasis, bullous pemphigoid and pemphigus vulgaris (Sadik et al., 2013 ▶). Based on the main theories on pathogenesis of PV, it seems that *C. fistula* fruit can be recommended for treatment of pemphigus erosions.

**Treatment**

Systemic glucocorticosteroids are still considered as the mainstay of medical therapy in pemphigus (Almugairen et al., 2013 ▶). Glucocorticosteroids include prednisolone dose regimen, pulsed dexamethasone as well as pulses of intravenous betamethasone or methylprednisolone. The guideline for management of PV by the British Association of Dermatologists recommends prescription of an initial 40-60 mg/day prednisolone in patients with mild disease and 60-100 mg/day in more severe cases (Kasperkiewicz et al., 2012 ▶).

Adjuvant drugs that are commonly used, increase the efficacy, and induce a steroid-sparing action, thereby, decreasing the maintenance dose of corticosteroids and consequently, corticosteroids side-effects (Harman et al., 2003 ▶). 

The efficacy of nicotinamide and pimecrolimus 1% as local treatments of pemphigus vulgaris has been studied in two separate trials. Nicotinamide, an anti-inflammatory drug in autoimmune-inflammatory diseases, could increase the epithelization index (Iraji and Banan, 2010 ▶). Pimecrolimus 1% which is a mast cell and T cell inhibitor was significantly more effective than placebo in improving the epithelization index of the participants (Iraji et al., 2010 ▶). The new topical medications including epidermal growth factor and a proteomics-derived desmoglein peptide are probably beneficial to pemphigus lesions (Ruocco et al., 2013a ▶).

Skin lesions are one of the most important sources of infection. Thus, any local treatment that could decrease the healing time of lesions and consequently reduce the total dosage of drugs, is favorable (Tabrizi et al., 2006 ▶).

***Cassia fistula***

*C. fistula, *commonly known as the Golden shower Indian Laburnum, is native to India, the Amazon and Sri Lanka (Neelam et al., 2011 ▶). *C. fistula *has been used for a long time both orally and topically for healing of wounds and burns in traditional medicine by the tribal communities of several countries (Ayyanar and Ignacimuthu, 2009 ▶).

 Black viscid pulp of *C. fistula *fruit is used to treat skin diseases and has been reported to possess anti-bacterial, anti-oxidant, and leukotriene inhibition properties (Shailajan et al., 2013 ▶).

 The fruits, stem, bark, and leaves of this plant contain a variety of biologically active compounds such as anthraquinones, flavonoids, flavon-3-ol derivatives, alkaloid, glycosides, tannin, saponin, triterpenoids, reducing sugar and steroids. Fruits of *C. fistula *contain rhein glycosides, fistulic acids, sennosides A B, all having various medicinal properties (Lee et al., 2001 ▶).

***C. fistula*** **L. and pemphigus**

*C. fistula* L. is a medicinal plant, which is widely used in TPM. It is cited as a treatment for related diseases in many Persian manuscripts. It was used orally for hot superficial and internal swelling (e.g. GI hot swelling) as gargle in a watery vehicle or topically in an oily vehicle of almond oil (i.e., *zomad*, a traditional lotion-like formulation) and is also mentioned as a mild laxative and purgative in TPM resources (Mozaffarpur et al., 2012 ▶) such as the Canon of Medicine (by Avicenna), Tohfatol Momenin (by Hakim Momen Tonekaboni) (Tonekaboni, 2007 ▶), Makhzan al-Advieh (by Aghili Shirazi) (Aghili Khorasani shirazi, 1388 ▶), Tibb al-Akbar (by Arzani) (Arzani, 1387 ▶), Exir-e-Azam (by Azamkan) (AzamKan, 1387 ▶), and Sharh-al-asbabvaAlamat (by Kermani) (Kermani, 1387 ▶). Moreover, *C. fistula* preparations are used as ethno-medicines in some other countries. For example, *C. fistula *fruit gel is used for the treatment of cutaneous leishmaniasis (Thirumal et al., 2012 ▶). A thick paste containing crushed leaves of* C. fistula *and coconut oil is prescribed for skin burn. Fruits are used as a cathartic remedy and to treat snake bites (Bhaleraoand Kelkar, 2012 ▶). The leaves and bark mixed with oil are applied to pustules and insect bites. The pulp of the fruit is also used as a dressing for gouty or rheumatic joint (Danish et al., 2011 ▶).

**Expected mechanisms of action of** ***C. fistula*** **fruit as a wound healing remedy in pemphigus vulgaris**

Animal studies have shown that both T and B cells with Dsg3 autoreactivity are critical elements in the pathogenesis of PV (Veldman et al., 2004 ▶). Strong evidence has shown that B cells are activated by Dsg3-specific auto-reactive T cells through IL-4 secretion (Okon and Werth, 2014 ▶).

1-The triterpen of *C. fistula* is lupeol (Thirumal et al., 2012 ▶). Lupeol is a naturally occurring lupine triterpene found in various plants (Geetha and Maralakshmi, 1999 ▶). Recently, lupeol was reported to exhibit significantly high wound healing potential in a dead space wound healing mouse model (Saleem, 2009 ▶). Lupeol was found to suppress various immune factors such as the phagocytic (cell-killing) activity of macrophages, and T-lymphocyte activity including CD4+ T helper cell-mediated cytokine generation. Administration of lupeol decreased CD4+ T cell counts and cytokines IL-2, IFN-γ (Th1) and IL-4 (Th2) levels (Bani et al., 2006 ▶; Siddique and Saleem, 2011 ▶). Serum TNFα has also been shown to correlate with disease activity, while anti-TNFα antibodies have been shown to inhibit PV acantholysis, *in vitro* (D’Auria et al., 1997 ▶; Feliciani et al., 2000 ▶). Lupeol treatment is also shown to decrease the generation of pro-inflammatory cytokines such as tumor necrosis factor α (TNFα) and Interleukin β (ILβ) in lipopolysaccharide-treated macrophages (Saleem, 2009 ▶).

 A comprehensive study showed that topical application of lupeol alleviated 12-0-tetradecanoylphorbol acetate (TPA)-induced inflammation in ear in a mouse model as it decreased the myeloperoxidase levels [neutrophil specific marker], thereby causing reduction in cell infiltration into inflamed tissues in mice (Fernández et al., 2001 ▶). The anti-inflammatory activity of water extract of the dried pulp of *C. fistula* L. was investigated using the carragenan-induced paw edema model in rats (Anwikar and Bhitre, 2010 ▶; Navanath et al., 2009 ▶).

2- Anthraquinone compounds encompass anti-inflammatory bioactivities whereas its natural derivative, rhein has been shown to effectively reduce tissue edema and free-radical production in rat models of inflammatory conditions (Tsang et al., 2013 ▶).

3- Flavonoids in *C. fistula* have shown anti-inflammatory effects by targeting reactive oxygen species (Anitha and Miruthula, 2014 ▶).

Fruit pulp powder of *C. fistula* was investigated for its antioxidant activity both *in vitro* and *in vivo*. High antioxidant activity of *C. fistula* may be due to its high phenolic and flavonoid content (Rajagopal et al., 2013 ▶). 

 The hydroalcoholic extract of the fruit pulp of* C. fistula* shows antioxidant activity by inhibiting DPPH and hydroxyl radical, total phenol content and reducing power activities (Bhalodia et al., 2013 ▶).

4- Anti-leukotriene effect of this herbal product has also been determined in previous studies. Recent data have shown that rhein has a strong potential to suppress the synthesis of some inflammation factors like leukotriene B4 andC4 in macrophages. It also represses the metabolism of arachidonic acid*. *In an* in vitro* study, rhein showed a protective activity in pancreatic cell injured by TNF-a (Guo et al., 2002 ▶).

The methanolic extract of *C. fistula *L. fruits blocked the 5-lipoxygenase catalysed formation of leukotriene B4 in bovine polymorphonuclear leukocytes (Rizvi et al., 2009 ▶).

5- *C. fistula *has also shown an anti-microbial activity in previous studies. 

Rhein, a natural anthraquinone derivative, which has antimicrobial and anticandidal properties is actively present in *C. fistula *fruit (Irshad et al., 2011 ▶). Hydroalcoholic and chloroform extracts of *C. fistula* fruit pulp were evaluated for potential antimicrobial activity. The antibacterial and antifungal activities of solvent extracts of *C. fistula* were tested against *Escherichia coli*, *Streptococcus pyogenes*, *Staphylococcus aureus*, *Pseudomonas aeruginosa*, and fungal strains *Aspergillus clavatus*, *Aspergillus niger*, *Candida albicans,* showing moderate to strong activity against most of the organisms tested (Bhalodia et al., 2012 ▶).

*C. fistula* has a widespread, low toxicity usage (Bahorun et al., 2004 ▶) when it is used in skin restoration.

Antioxidants play a determining role in progression of wound healing. In addition, anti-inflammatory agents play a key role in the wound healing process and prevent exacerbation of wound conditions. Antimicrobial agents are also useful in management of microbial infections which may concomitantly occur in severe and chronic wounds (Farzaei et al., 2014 ▶).

*C. fistula* fruit oil has lupeol, anthraquinone compounds like rhein and flavonoids.

Since anthraquinone, triterpenes and flavonoids are oil soluble, it is likely that these chemicals are present in our product; hence, future studies should include chemical analysis of the cassia oil, to ensure about the presence of these chemicals.

Lupeol with a high potential of wound healing, suppression of CD4+ T helper cell, and decreasing the generation of pro-inflammatory cytokine (IL2, IL4**, **TNF, IFN) has shown anti-inflammatory effect and inhibited Dsg3 autoreactivity, which are considered as the critical elements in the pathogenesis of PV.

Rhein which effectively reduced free-radical production has also shown antioxidant, antimicrobial, and anticandidal activities. Rhein has a strong potential to suppress the synthesis of some inflammation factors like leukotriene B4 andC4, in macrophages. High antioxidant activity of *C. fistula* may be related to its high phenolic and flavonoid content**. **Based on the above-described mechanisms of action, it is expected that *C. fistula* fruit oil is an effective formulation for wound healing of pemphigus erosions. *C. fistula* has a widespread use, and low toxicity; thus, it has no limitation in usage.
